# Blebbistatin Inhibits Neomycin-Induced Apoptosis in Hair Cell-Like HEI-OC-1 Cells and in Cochlear Hair Cells

**DOI:** 10.3389/fncel.2019.00590

**Published:** 2020-02-05

**Authors:** Song Gao, Cheng Cheng, Maohua Wang, Pei Jiang, Liyan Zhang, Ya Wang, Huihui Wu, Xuanfu Zeng, Hui Wang, Xia Gao, Yongming Ma, Renjie Chai

**Affiliations:** ^1^Department of Otolaryngology, Affiliated People’s Hospital of Jiangsu University, Zhenjiang, China; ^2^Department of Otolaryngology Head and Neck Surgery, Nanjing Drum Tower Hospital Clinical College of Nanjing Medical University, Jiangsu Provincial Key Medical Discipline (Laboratory), Nanjing, China; ^3^Research Institute of Otolaryngology, Nanjing, China; ^4^Department of Otolaryngology, Head and Neck Surgery, XiangYa School of Medicine, Central South University, Changsha, China; ^5^MOE Key Laboratory for Developmental Genes and Human Disease, Institute of Life Sciences, Southeast University, Nanjing, China; ^6^Department of Otolaryngology Head and Neck Surgery, Shanghai Jiao Tong University Affiliated Sixth People’s Hospital, Shanghai, China; ^7^Co-Innovation Center of Neuroregeneration, Nantong University, Nantong, China; ^8^Institute for Stem Cell and Regeneration, Chinese Academy of Science, Beijing, China; ^9^Beijing Key Laboratory of Neural Regeneration and Repair, Capital Medical University, Beijing, China; ^10^Jiangsu Province High-Tech Key Laboratory for Bio-Medical Research, Southeast University, Nanjing, China

**Keywords:** aminoglycoside, hair cell, blebbistatin, apoptosis, ROS, hearing, synaptic plasticity

## Abstract

Aging, noise, and ototoxic drug-induced hair cell (HC) loss are the major causes of sensorineural hearing loss. Aminoglycoside antibiotics are commonly used in the clinic, but these often have ototoxic side effects due to the accumulation of oxygen-free radicals and the subsequent induction of HC apoptosis. Blebbistatin is a myosin II inhibitor that regulates microtubule assembly and myosin–actin interactions, and most research has focused on its ability to modulate cardiac or urinary bladder contractility. By regulating the cytoskeletal structure and reducing the accumulation of reactive oxygen species (ROS), blebbistatin can prevent apoptosis in many different types of cells. However, there are no reports on the effect of blebbistatin in HC apoptosis. In this study, we found that the presence of blebbistatin significantly inhibited neomycin-induced apoptosis in HC-like HEI-OC-1 cells. We also found that blebbistatin treatment significantly increased the mitochondrial membrane potential (MMP), decreased ROS accumulation, and inhibited pro-apoptotic gene expression in both HC-like HEI-OC-1 cells and explant-cultured cochlear HCs after neomycin exposure. Meanwhile, blebbistatin can protect the synaptic connections between HCs and cochlear spiral ganglion neurons. This study showed that blebbistatin could maintain mitochondrial function and reduce the ROS level and thus could maintain the viability of HCs after neomycin exposure and the neural function in the inner ear, suggesting that blebbistatin has potential clinic application in protecting against ototoxic drug-induced HC loss.

## Introduction

Aging, noise, and ototoxic drugs are major causes of hair cell (HC) damage leading to sensorineural hearing loss. Aminoglycoside antibiotics are widely used against gram-negative bacterial infections because of their low cost and effectiveness (Becker and Cooper, 2013), but these drugs can cause HC loss by activating the apoptosis pathway (Jiang et al., 2017). Thus, it is important to find an effective way to reduce the ototoxicity of aminoglycosides. Aminoglycoside antibiotics mainly accumulate in the lysosomes and mitochondria in HCs, inducing the accumulation of intracellular reactive oxygen species (ROS), which in turn induce apoptosis and are the major factor leading to HC damage (Coffin et al., 2013; Liu et al., 2016, 2019; Wang et al., 2016; He et al., 2017; Waqas et al., 2017; Yu et al., 2017; Li A. et al., 2018; Li H. et al., 2018). Several studies have shown that ischemia–reperfusion-induced deafness, noise-induced deafness, presbycusis, and ototoxic drug-induced deafness are all closely related to the accumulation of ROS in HCs (Seidman et al., 2000; Sena and Chandel, 2012).

Many studies, including our previous studies, have shown that caspase-associated apoptosis plays an important role in aminoglycoside-induced ototoxicity (Guan et al., 2016; He et al., 2017). The accumulation of ROS in the lysosomes and mitochondria of HCs leads to the upregulation of caspase genes, which further induces apoptosis of HCs (Esterberg et al., 2016; Jiang et al., 2016; Guo et al., 2019). In the central nervous system, myosin contraction plays an important role in oxidative stress-related neuronal apoptosis, myosin contraction, and the formation of relevant complexes needed to activate the expression of caspase-3 through the ROCK1-related pathway, indicating positive feedback regulation between myosin contraction and the oxidative stress-induced apoptosis pathway (Wang et al., 2017). However, the role of myosin contraction in cochlear HC apoptosis remains uninvestigated.

Blebbistatin is a myosin II inhibitor, and it interferes with myosin–actin interactions and microtubule assembly (Kovács et al., 2004). It has been reported that blebbistatin reduces apoptosis in neurons, cardiomyocytes, and erythrocytes, and by inhibiting myosin IIA–actin interactions, blebbistatin increases mitochondrial length and reduces calcium overload, reduces damage from oxygen free radicals and mitochondrial dysfunction, and decreases caspase activity (Lang et al., 2011; Wang et al., 2017; Li F. et al., 2018). Many research has focused on blebbistatin’s effects on cellular morphology or modulating cardiac contractility (Chen et al., 2018), and the role of blebbistatin in protecting against aminoglycoside-induced HC apoptosis has not been investigated yet.

In this study, we found that blebbistatin significantly reduced ROS accumulation and maintained mitochondrial function and thus prevented neomycin-induced apoptosis in HEI-OC-1 cells and explant-cultured cochlear HCs *in vitro*. Our results suggest that blebbistatin might serve as a new therapeutic drug for the prevention of aminoglycoside-induced HC loss.

## Materials and Methods

### Animals

All animal procedures were performed according to protocols approved by the Animal Care and Use Committee of Southeast University. All efforts were made to use minimal animals and to prevent their suffering.

### Whole Organ Explant Culture

Cochlear sensory epithelium was dissected from postnatal day (P)3 wild-type FVB mice and cultured in DMEM/F12 (Gibco, Gaithersburg, MD, USA, 11330-032) supplemented with 2% B27 (Invitrogen, Waltham, MA, USA, 17504044), 1% N-2 (Invitrogen, Waltham, MA, USA, 17502-048), and 50 μg/ml ampicillin (Sigma–Aldrich, St. Louis, MO, USA, P0781). In the experimental group, the cochleae were treated with 0.5 mM neomycin (Sigma–Aldrich, St. Louis, MO, USA, N6386-5G) and 1 μM blebbistatin (dissolved in DMSO, Boehringer Ingelheim Pharma GmbH, Biberach an der Riß, Germany) for 12 h and allowed to recover for another 12 h. Equivalent amounts of DMSO (Sigma–Aldrich, St. Louis, MO, USA, D8371) were added to the control and neomycin-only groups. The tissues were cultured at 37°C with 5% CO_2_.

### Cell Culture

HEI-OC-1 cells were divided into three groups and cultured in DMEM (Corning, Corning city, NY, USA, 10-013-CVC) supplemented with 10% FBS (Pansera, P30-2602) and 50 μg/ml ampicillin for 12 h. After this initial incubation, the experimental group was treated with 2 mM neomycin and 0.01 μM to 5 μM blebbistatin in 6-well plates, while the neomycin-only group was treated with 2 mM neomycin and an equivalent volume of DMSO in place of the blebbistatin. After another 24 h of culture, the cells were thoroughly washed with PBS and cultured in DMEM with ampicillin for an additional 12 h recovery. Control cells without neomycin or blebbistatin were treated with an equivalent volume of DMSO and incubated under identical conditions. Finally, the cells were imaged with an inverted phase-contrast microscope.

### CCK-8 Assay

Cell death was measured using the Cell Counting CCK-8 Kit (Protein Biotechnology, CC201-01). Briefly, HEI-OC-1 cells were exposed to 2 mM neomycin in 96-well plates for 12 h. After removing the neomycin, the tissues were allowed to recover for another 12 h. Blebbistatin was added throughout the entire process in the experimental group, and an equivalent volume of DMSO was added in the neomycin-only group. All cells were then incubated with 10 μl of CCK-8 in each well for 30 min at 37°C, and a microtiter plate reader (Bio-Rad) was used to measure the optical densities at 450 nm.

### Immunofluorescence

The antibodies and staining kits used in this article included the Live-Dead Cell Staining Kit (Biovision, Milpitas, CA, USA, K501-100), stained with fluorescein diacetate (FDA, green) and propidium iodide (PI, red), TUNEL BrightRed Apoptosis Detection Kit (Vazyme, A113-01), anti-cleaved-caspase-3 antibody (Cell Signaling Technology, Danvers, MA, USA, 9664S), Mito-SOX Red (Life Technologies, Carlsbad, CA, USA, M36008), anti-Ctbp2 (BD Biosciences, San Jose, CA, USA, 612044), anti-myosin7a antibody (Proteus Bioscience, #25-6790, 1:1,000 dilution), Alexa Fluor 647 donkey anti-goat IgG (H + L; Invitrogen, Waltham, MA, USA, A-21447, 1:500 dilution), Alexa Fluor 555 donkey anti-rabbit IgG (H + L; Invitrogen, Waltham, MA, USA, A-31572, 1:500 dilution), and DAPI (Solarbio, C0060, 1:1,000 dilution).

Except for the staining kits, the cells or tissues were incubated with 4% paraformaldehyde (Sigma–Aldrich, St. Louis, MO, USA, 158127) for 1 h and then washed three times with PBST [1× PBS with 0.1% Triton X-100 (Solarbio, 1109F0521)] and incubated for 1 h in blocking medium (PBS with 10% heat-inactivated donkey serum, 1% Triton X-100, 1% BSA, and 0.02% sodium azide at pH 7.2) at room temperature. The samples were marked with primary antibody diluted in PBT-1 (PBS with 10% Triton X-100, 5% heat-inactivated donkey serum, 1% BSA, and 0.02% sodium azide at pH 7.2) for 8 h at 4°C. After washing three times with PBST, the samples were marked with the secondary antibody diluted in PBT-2 (PBS with 1% BSA and 0.1% Triton X-100 at pH 7.2) for 1 h. The samples were washed again three times with PBST and mounted on slides. The samples were imaged with an LSM700 confocal microscope.

### Flow Cytometry

An Annexin V-FITC Kit (Beyotime, C1062) was used to detect the apoptotic cells. For determining mitochondrial membrane potential (MMP) and analyzing ROS production, cells were stained using the TMRE Mitochondrial Membrane Potential Assay Kit (Abcam, Cambridge, UK, ab113852) and MitoTracker^®^ Red CMXRos (Yeasen, 40741ES50) and analyzed by flow cytometry (FACSCanto, BD) within 1 h.

### PCR

RNA was extracted using TRIzol reagent (Protein Biotechnology), and cDNA was reversed transcribed with the RevertAid First Strand cDNA synthesis kit (Thermo Fisher Scientific, Waltham, MA, USA). For quantitative polymerase chain reaction (qPCR), SYBR Green (Roche, Basel, Switzerland, 17747200) was used on a real-time PCR apparatus (CFX96, Bio-Rad). *Gapdh* was used as the reference endogenous gene.

**Table:** 

Gene	Forward sequence (5′–3′	Reverse sequence (5′–3′
*Gapdh*	AGGTCGGTGTGAACGGATTTG	TGTAGACCATGTAGTTGPAGGTCA
*Bax*	TGAAGACAGGGGCCTTTTTG	AATTCGCCGGAGACACTCG
*Casp3*	ATGGAGAACAACAAAACCTCAGT	TTGCTCCCATGTATGGTCTTTAC
*NF-κB*	ATGGCAGATGATCCCTAC	TGTTGACAGTGGTATTTCTGGTG
*Bcl-2*	ATGCTTTGTGGAACTATATGGC	GGTATGCACCCAGAGTGATGC
*Gsr*	TGCACTTCCCGGTAGGAAAC	GATCGCAACTGGGGTGAGAA
*Sodl*	AACCAGTTGTGTTGTCAGGAC	CCACCATGTTTCTTAGAGTGAGG
*Nqo1*	AGGATGGGAGGTACTCGAATC	AGGCGTCCTTCCTTATATGCTA
*Alox15*	GGCTCCAACAACGAGGTCTAC	AGGTATTCTGACACATCCACCTT

### Statistical Analysis

All experiments were repeated at least three times, and the data are shown as the mean ± SD. All statistical analyses were conducted using Microsoft Excel and GraphPad Prism 7. Two-tailed, unpaired student’s *t*-tests were used to determine statistical significance when comparing two groups, and one-way ANOVA followed by a Dunnett multiple comparisons test was used when comparing more than two groups. *P* < 0.05 was considered statistically significant.

## Results

### Blebbistatin Treatment Significantly Increased the Viability of HC-Like HEI-OC-1 Cells After Neomycin Exposure

**Figure 1:**
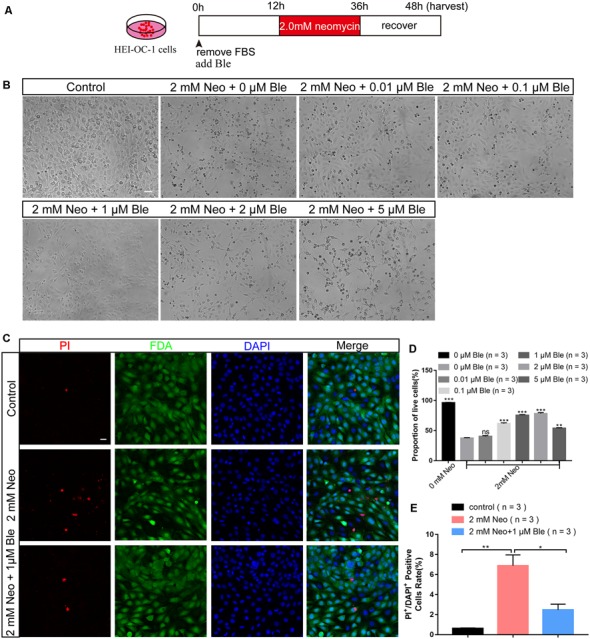
Blebbistatin significantly enhanced the viability of HEI-OC-1 cells after neomycin exposure. **(A)** Schematic diagram of blebbistatin (Ble) and neomycin addition in cell culture. **(B)** The survival of hair cell (HC)-like HEI-OC-1 cells cultured under the same conditions with different concentrations of blebbistatin. Scale bars = 100 μm. **(C)** Images of HEI-OC-1 cells stained with FDA (green) and PI (red). Scale bars = 20 μm. **(D)** The result of the CCK-8 assay. **(E)** The proportions of live and dead cells in **(D)**. *p < 0.05, **p < 0.01, ***p < 0.001, ns, no significant.

To determine the protective effect of blebbistatin in HC-like HEI-OC-1 cells, the cells were pre-treated with different doses of blebbistatin for 12 h before neomycin exposure. We then treated the HEI-OC-1 cells with 2 mM neomycin together with blebbistatin for 24 h and measured the survival of HEI-OC-1 cells using the CCK-8 kit (Figure 1A). Survival decreased significantly after 2 mM neomycin exposure, and blebbistatin protected against neomycin-induced cell death (Figures 1B,C). The CCK-8 results showed that the viability gradually increased with low concentrations of blebbistatin, but once the concentration of blebbistatin was higher than 2 μM, the viability of HEI-OC-1 cells began to decrease (Figure 1D). Cell morphology was significantly altered with 2 μM blebbistatin (Figure 1B), so we chose 1 μM blebbistatin pre-treatment for 12 h as the treatment condition in the rest of this study. To confirm this finding, we measured the percentage of live and dead cells in the control group, neomycin-only group, and blebbistatin group using the live-dead cell staining kit. Blebbistatin treatment significantly reduced cell death caused by neomycin exposure (Figures 1C,E). At the same time, we used myosin7a to label the HEI-OC-1 cells and found that compared with the neomycin-only group, living cells morphology in blebbistatin group is more similar to the control group (Supplementary Figure S1).

### Blebbistatin Treatment Reduced Neomycin-Induced Cochlear HC Loss in Whole-Organ Explant Cultures *in vitro*

**Figure 2:**
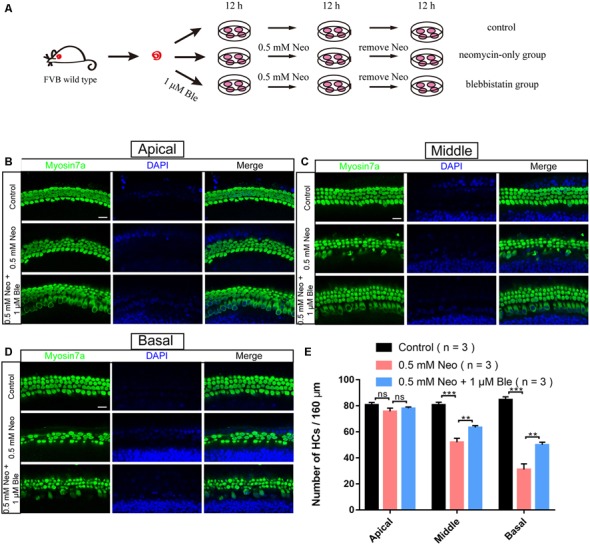
Blebbistatin promoted HC survival in the cochlea after neomycin exposure. **(A)** Schematic diagram of drug addition in tissue culture. **(B–D)** HCs in the apical **(B)**, middle **(C)**, and basal turns **(D)** of the cochlea were stained with anti-myosin7a antibody in the control, 0.5 mM neomycin, and 0.5 mM neomycin + 1 μM blebbistatin groups. **(E)** Quantification of the number of myosin7a-positive cells in the apical, middle, and basal turns of the cochlea. **p < 0.01, ***p < 0.001, ns, no significant. Scale bars = 16 μm.

To determine the effect of blebbistatin in protecting cochlear HCs after neomycin damage, we used whole-organ explant cultures. Consistent with our previous studies (Guan et al., 2016; He et al., 2017), we dissected the cochleae from P3 wild-type mice, and the cultured cochleae were pre-treated with 1 μM blebbistatin for 12 h before neomycin damage. We then treated the cultured cochleae with 0.5 mM neomycin together with blebbistatin for 12 h (Figure 2A). Consistent with the results in HEI-OC-1 cells, immunofluorescence staining with a myosin7a antibody and DAPI showed that the myosin7a-positive HC numbers of the middle and basal turns of the cochlea were significantly decreased after neomycin exposure, while blebbistatin treatment significantly increased the HC number compared to the neomycin-only group (Figures 2B–D). Consistent with previous reports (Li A. et al., 2018), the neomycin-induced HC loss was mainly in the middle and basal turns of the cochlea, and no significant differences were seen in the apical turn in the blebbistatin group, neomycin-only group, and control group (Figure 2E).

### Blebbistatin Treatment Significantly Decreased Apoptosis in HEI-OC-1 Cells After Neomycin Exposure

**Figure 3:**
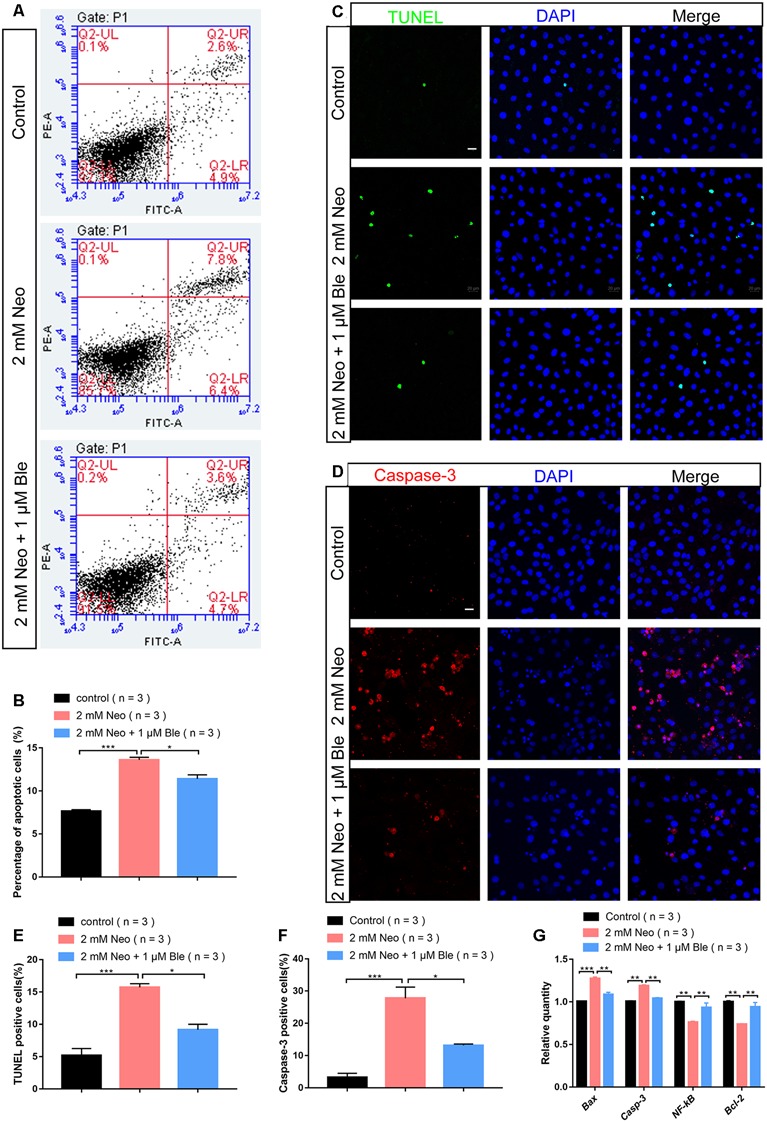
Blebbistatin reduced neomycin-induced apoptosis in HEI-OC-1 cells. **(A)** TUNEL staining showing the apoptotic HEI-OC-1 cells after different treatments. The TUNEL-positive apoptotic cells increased in the neomycin-only group compared with the controls and decreased in the 2 mM neomycin + 1 μM blebbistatin group compared with the neomycin-only group. **(B)** Cleaved-caspase-3 and DAPI double staining showing the apoptotic HEI-OC-1 cells after the different treatments. **(C)** Apoptosis analysis by flow cytometry after different treatments. **(D)** Quantification of the flow cytometry results. **(E)** Quantification of the numbers of TUNEL/DAPI double-positive cells in panel (**A**). **(F)** Quantification of the numbers of Caspase-3/DAPI double-positive cells in panel (**B**). **(G)** Quantitative polymerase chain reaction (qPCR) results showing the expression of pro-apoptotic factors like *caspase-3* and *Bax* and anti-apoptotic factors like *Bcl-2* and *NF-kB* after neomycin and blebbistatin treatment. *p < 0.05, **p < 0.01, ***p < 0.001. Scale bars = 20 μm.

To determine the effect of blebbistatin on HEI-OC-1 cell apoptosis after neomycin exposure, we measured the percentage of cell death and cell apoptosis using flow cytometry. We used propidium iodide to label the dead cells and Annexin V to label the cells undergoing apoptosis and showed that the cells pre-treated with 1 μM blebbistatin had a significantly lower rate of apoptosis compared to the neomycin-only group (Figures 3A,B).

To confirm the effect of blebbistatin on inhibiting HEI-OC-1 cell apoptosis, we used TUNEL staining and a cleaved-caspase-3 antibody. The numbers of both TUNEL-positive and cleaved-caspase-3-positive cells in the neomycin-only group were significantly greater than the control group (Figures 3C,D), while pre-treatment with 1 μM blebbistatin significantly reduced the proportions of TUNEL-positive and cleaved-caspase-3-positive cells after neomycin exposure (Figures 3E,F).

Our qPCR results also showed that the expression of pro-apoptotic genes like *Casp3* and *Bax* were significantly decreased in HEI-OC-1 cells pre-treated with 1 μM blebbistatin, while the expression of the anti-apoptotic genes *Bcl-2* and *NF-kB* were significantly increased in the blebbistatin pre-treated group compared to the neomycin-only group (Figure 3G). Together, our results suggest that blebbistatin protects HEI-OC-1 cells against neomycin exposure by inhibiting neomycin-induced apoptosis.

### Blebbistatin Treatment Reduced Neomycin-Induced Apoptosis of Cochlear HCs in Whole-Organ Explant Cultures *in vitro*

**Figure 4:**
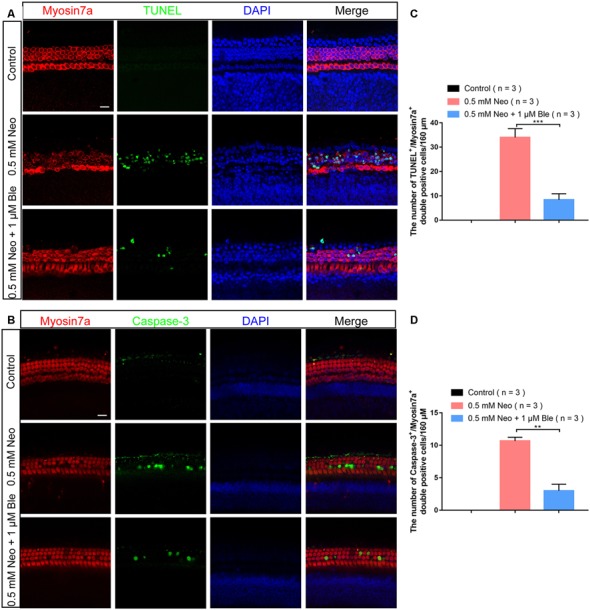
Neomycin-induced HC apoptosis decreased after treatment with blebbistatin. **(A)** The number of TUNEL-positive cells decreased in the 0.5 mM neomycin + 1 μM blebbistatin group compared with the neomycin-only group (middle turns). **(B)** The number of cleaved caspase-3-positive cells decreased in the 0.5 mM neomycin + 1 μM blebbistatin group compared with the neomycin-only group (middle turns). **(C)** Quantification of the numbers of TUNEL/myosin7a double-positive cells in panel (**A**). **(D)** Quantification of the numbers of cleaved caspase-3/myosin7a double-positive cells in panel (**B**). **p < 0.01, ***p < 0.001. Scale bars = 16 μm.

To verify the effects of blebbistatin on neomycin-induced HC loss in whole-organ explant cultures, we also stained the explant-cultured cochleae for TUNEL and cleaved-caspase-3 after blebbistatin treatment and neomycin exposure. Consistent with the results in HEI-OC-1 cells, the numbers of both TUNEL-positive and cleaved-caspase-3–positive HCs were significantly increased in the neomycin-only group compared to the control group (Figures 4A–D). Blebbistatin treatment reduced neomycin-induced HC apoptosis, and the proportions of TUNEL-positive and cleaved-caspase-3-positive HCs were significantly lower after pre-treatment with 1 μM blebbistatin (Figures 4A–D).

### Blebbistatin Treatment Significantly Increased the MMP of HEI-OC-1 Cells After Neomycin Exposure

**Figure 5:**
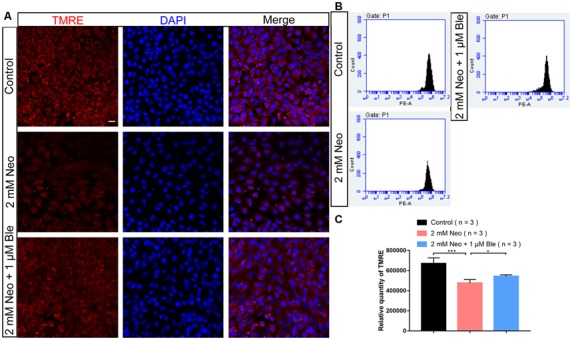
Blebbistatin maintains the mitochondrial membrane potential (MMP) after neomycin exposure. **(A)** HEI-OC-1 cells were labeled using the TMRE staining kit. **(B)** The analysis of MMP by flow cytometry showing that the TMRE intensity was reduced after 2 mM neomycin treatment for 24 h compared with the undamaged controls and that the TMRE intensity in the 2 mM neomycin + 1 μM blebbistatin group was increased significantly compared with the neomycin-only group. **(C)** Quantification of the data in panel **(B)**. *p < 0.05, ***p < 0.001. Scale bars = 20 μm.

Mitochondria are the main site of cellular ROS production, and the production of ROS occurs mainly in the mitochondrial oxidative respiratory chain. Thus, mitochondrial structural and functional disorders can lead to mitochondrial ROS accumulation, which is the main inducer of apoptosis (Liu and Yan, 2007). To investigate the mechanism through which blebbistatin prevents neomycin-induced apoptosis, the TMRE kit was used to evaluate changes in the MMP of HEI-OC-1 cells using flow cytometry analysis and immunofluorescence staining (Samudio et al., 2005). The MMP of HEI-OC-1 cells was significantly lower after 2 mM neomycin exposure, while HEI-OC-1 cells pre-treated with 1 μM blebbistatin had significantly greater TMRE intensity compared to the neomycin-only group (Figures 5A–C). These results suggest that blebbistatin prevents neomycin-induced mitochondrial dysfunction in HEI-OC-1 cells.

### Blebbistatin Treatment Significantly Attenuated Neomycin-Induced Oxidative Stress in HEI-OC-1 Cells

**Figure 6:**
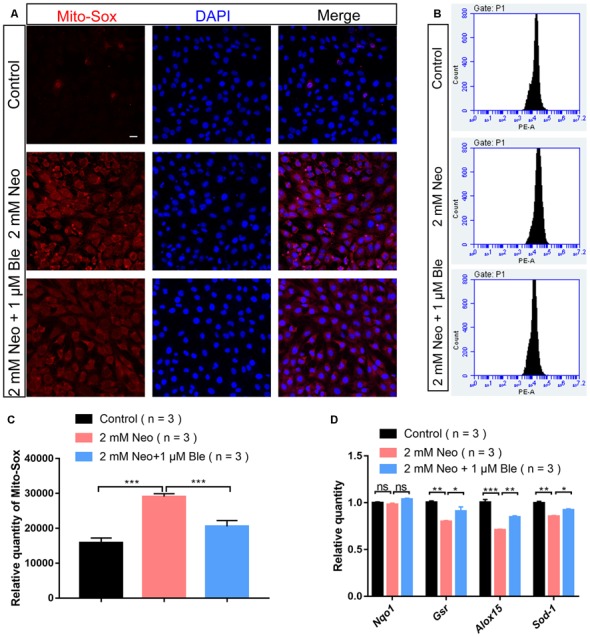
Blebbistatin decreased reactive oxygen species (ROS) levels in HEI-OC-1 cells after neomycin injury. **(A)** The immunofluorescence intensity of Mito-SOX was increased after 2 mM neomycin treatment for 24 h compared with the undamaged controls, and Mito-SOX intensity was significantly reduced in the 2 mM neomycin + 1 μM blebbistatin group compared with the neomycin-only group. Scale bars = 20 μm. **(B)** Flow cytometry data showing the intensity of Mito-SOX in the control, neomycin-only, and 2 mM neomycin + 1 μM blebbistatin groups. **(C)** Quantification of the data in panel (**B**). **(D)** qPCR results showing the expression of the antioxidant genes *Gsr, Sod1, Alox15*, and *Nqo1* after neomycin and blebbistatin treatment. *p < 0.05, **p < 0.01, ***p < 0.001, ns, no significant. Scale bars = 16 μm.

Recent studies have demonstrated that the production of ROS by the mitochondria is the major cause of aminoglycoside-induced HC apoptosis (Huang et al., 2000; Balaban et al., 2005). Mito-SOX Red has been reported to selectively detect mitochondrial superoxide (Kalyanaraman et al., 2017), and here we also used Mito-SOX Red to detect mitochondrial ROS levels in HEI-OC-1 cells by immunofluorescence staining and flow cytometry analysis. Neomycin exposure significantly increased the ROS levels, while the HEI-OC-1 cells pre-treated with 1 μM blebbistatin had significantly lower ROS levels compared with the neomycin-only cells (Figures 6A–C).

To confirm this result, the expression levels of oxidant genes were also measured using qPCR. We found that after neomycin exposure, the HEI-OC-1 cells pre-treated with 1 μM blebbistatin had significantly higher expression levels of the antioxidant genes *Gsr*, *Alox15*, and *Sod1* compared to the neomycin-only cells (Figure 6D). Together, our data demonstrated that blebbistatin treatment significantly increased antioxidant gene expression and prevented the accumulation of mitochondrial ROS in HEI-OC-1 cells and thus protected HEI-OC-1 cells against neomycin-induced apoptosis.

### Blebbistatin Treatment Significantly Reduced Neomycin-Induced Oxidative Stress in Cultured Cochlear HCs

**Figure 7:**
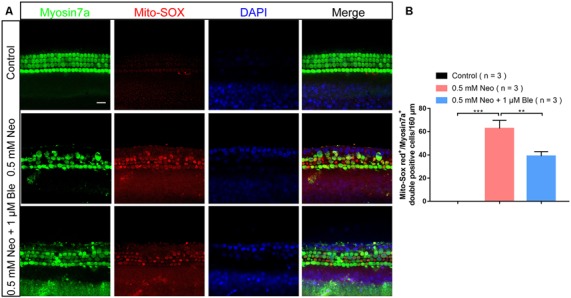
Blebbistatin decreased ROS levels in cochlear HCs after neomycin injury. **(A)** The immunofluorescence intensity of Mito-SOX was increased after 0.5 mM neomycin treatment for 12 h compared with the undamaged controls, and Mito-SOX intensity was significantly reduced in the 0.5 mM neomycin + 1 μM blebbistatin group compared with the neomycin-only group. **(B)** Quantification of the numbers of Mito-SOX/myosin7a double-positive cells in A. **p < 0.01, ***p < 0.001. Scale bars = 16 μm.

To determine the protective mechanism of blebbistatin in cochlear HCs in whole-organ explant cultures, we also stained the cultured cochleae with Mito-SOX Red to detect mitochondrial ROS levels in HCs after blebbistatin treatment and neomycin damage. Consistent with the results in HEI-OC-1 cells, we found that neomycin exposure significantly increased HC loss and ROS levels, while blebbistatin treatment significantly reduced the HC loss and ROS levels compared to the neomycin-only group (Figures 7A,B).

### Blebbistatin Protects the Synapses Between Hair Cells and Spiral Ganglion Neurons

**Figure 8:**
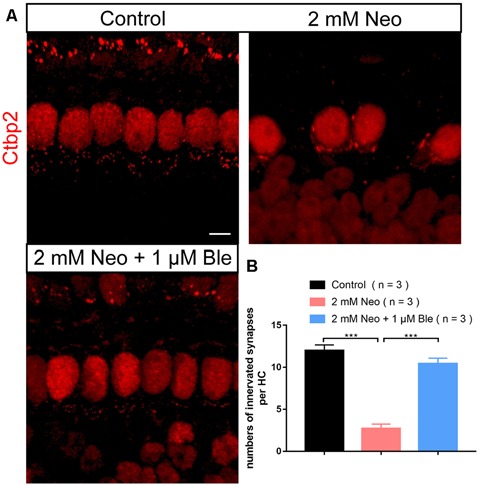
Pre-synapse staining of the HCs. **(A)** The presynaptic marker Ctbp2 was used to label the ribbon synapses on HCs. **(B)** Quantification of the presynaptic number in HCs. ***p < 0.001. Scale bar = 5 μm.

To confirm whether blebbistatin can protect the synapses between HCs and cochlear spiral ganglion neurons, we used Ctbp2 to label the synapses of the HCs and found that the number of HCs’ synapse decreased significantly in the neomycin group compared with the blebbistatin group and the control group (Figures 8A,B). These results suggest that blebbistatin prevents neomycin-induced synaptic damage in HCs.

## Discussion

Ototoxic side effects limit the clinical application of aminoglycoside antibiotics (Durante-Mangoni et al., 2009; Zimmerman and Lahav, 2013). Aminoglycosides can produce a large number of highly toxic ROS, and this occurs mainly in the organ of Corti, which is the main sensory organ for hearing (Nadol, 1993). Under physiological conditions, the ROS produced by mitochondrial metabolism are removed by the antioxidant mechanisms of the HC. However, aminoglycoside exposure increases ROS production in the cochlear HCs, and the excess ROS overwhelm the cellular defense mechanisms and eventually trigger apoptosis in HCs (Chen et al., 2015; Quan et al., 2015; Esterberg et al., 2016). Therefore, finding effective ways to reduce cellular ROS production in HCs is the key to preventing aminoglycoside-induced ototoxicity and is a main focus in the hearing research field.

Blebbistatin is a myosin II inhibitor, and it regulates microtubule assembly and myosin–actin interactions. The activity of blebbistatin on the cytoskeleton has been shown to be involved in the regulation of cell structure, morphology (Yoon et al., 2019), and migration (Hu et al., 2019; Wang et al., 2019) and to maintain the survival and growth of stem cells (Zhao et al., 2015) and to reduce oxidative stress-induced apoptosis. Blebbistatin protects Lgr5+ stem cells against colitis-induced epithelium injury in gastrointestinal tissues through the Myh9-Rac1-PAK1-Akt pathway (Zhao et al., 2015) and induces cell migration through myosin-II-related matrix stretch and recoil (Vicente-Manzanares et al., 2009). Blebbistatin has also been reported to inhibit apoptosis by reducing the accumulation of ROS in neuronal tissues (Wang et al., 2017). The interconnections and differences in these effects in various cells and tissues are also of interest and need to be explored further.

To explore the effects of blebbistatin in protecting against neomycin-induced damage, we used HC-like HEI-OC-1 cells and cochlear whole-organ explant cultures *in vitro*. Results in both systems showed that blebbistatin significantly reduced mitochondrial ROS accumulation and inhibited cell apoptosis, thus preventing the neomycin-induced apoptotic cell death of HEI-OC-1 cells and cultured cochlear HCs. Cochlear whole-organ explant cultures showed that blebbistatin protected against neomycin-induced HC loss in the middle and basal turns of the cochlea, while the damage in the apical turn was only mild and thus no protection by blebbistatin was observed. This was because aminoglycosides are preferentially localized at the base of cochlea, and both the aminoglycoside concentration and the extent of HC damage form a decreasing gradient from the base to the apex (Karasawa et al., 2008; Marcotti et al., 2010). Moreover, aminoglycoside-induced hearing loss also shows a decreasing gradient from high frequency to low frequency, which is consistent with the HC damage occurring primarily in the base and decreasing towards the apex (Chen et al., 2015; Guo et al., 2019). Because basal HCs are more sensitive to neomycin, we have paid the most attention to the protection of HCs in the basal turn.

We also found that the protective effect of blebbistatin is dose dependent, and 1 μM blebbistatin had significantly greater protective effects than 0.1 μM blebbistatin (Figures 1A–D). A similar level of protection was seen for 2 μM blebbistatin, but at 5 μM, the protective effect began to decline, suggesting that high concentrations of blebbistatin might damage HCs. Previous studies also showed that at concentrations higher than 1 μM, blebbistatin shows toxic effects in cardiomyocytes (Li F. et al., 2018). Also, in the concentration range of 0.5–5 μM, blebbistatin preferentially blocks the connection between myosin and actin (Kovács et al., 2004), and in neurons, 1 μM blebbistatin treatment maintains the cellular structure after H_2_O_2_ exposure (Wang et al., 2017). Thus, in this study we used 1 μM blebbistatin in both the HC-like HEI-OC-1 cells and the cochlear whole-organ explant cultures.

ROS accumulation and mitochondrial dysfunction have been reported to be involved in HC apoptosis (Hu et al., 2008; He et al., 2016). ROS accumulation leads to mitochondrial depolarization, which results in a decrease in the MMP and the subsequent release of apoptotic factors. Under physiological conditions, spontaneous generation and elimination keep the ROS level stable, and this is regulated by numerous antioxidant and oxidant genes. This study found that blebbistatin significantly increased the expression of the antioxidant genes *Gsr*, *Sod1*, and *Alox15* after neomycin exposure, which significantly decreased the ROS levels and increased the MMP in HEI-OC-1 cells. Our results suggest that blebbistatin is an effective drug in reducing the ROS level and maintaining mitochondrial function after neomycin exposure. This is consistent with observations in myocardial and other nucleated cells that blebbistatin protects mitochondrial function by stabilizing the morphology of the cytoskeleton and that it reduces ROS accumulation in order to prevent apoptosis (Lang et al., 2011; Wang et al., 2017; Li F. et al., 2018; Miura et al., 2018).

Regarding plasticity and reconstruction of neural network after hearing injury, Ctpb2 was usually used to label the synapse, and the number of innervated synapses was compared to assess the structure and function of neural connections (Zhang et al., 2019). Our results suggest that blebbistatin has a good protective effect on synaptic damage caused by neomycin in HCs.

Compared to other drugs known to reduce HC damage caused by aminoglycosides, we think that blebbistatin can reduce the accumulation of ROS more stably and efficiently. But it has to be pointed out that high concentrations of blebbistatin correspond to the low efficiency of treatment and change the normal shape of cells to some extent. Drug dosage is essential to be fully considered for future animal or human experiments.

In conclusion, our results suggest that blebbistatin can maintain the balance of oxidant and anti-oxidant gene expression and reduce the accumulation of ROS and thus maintain mitochondrial function and prevent apoptosis in HEI-OC-1 cells and cultured cochlear HCs after neomycin exposure. These results suggest that blebbistatin might have potential clinical application in preventing aminoglycoside-induced HC loss and subsequent hearing loss, and we will further investigate its protective mechanism and clinical application in future studies.

## Data Availability Statement

The raw data supporting the conclusions of this article will be made available by the authors, without undue reservation, to any qualified researcher.

## Ethics Statement

The animal study was reviewed and approved by Animal Care and Use Committee of Southeast University.

## Author Contributions

All authors listed have made a substantial, direct and intellectual contribution to the work, and approved it for publication.

## Conflict of Interest

The authors declare that the research was conducted in the absence of any commercial or financial relationships that could be construed as a potential conflict of interest.

## References

[B34] Balaban R. S. Nemoto S. Finkel T. (2005). Mitochondria, oxidants, and aging. Cell 120, 483–495. 10.1016/j.cell.2005.02.001 15734681

[B2] Becker B. Cooper M. A. (2013). Aminoglycoside antibiotics in the 21st century. ACS Chem. Biol. 8, 105–115. 10.1021/cb3005116 23110460

[B5] Chen Y. Li L. Ni W. Zhang Y. Sun S. Miao D.. (2015). Bmi1 regulates auditory hair cell survival by maintaining redox balance. Cell Death Dis. 6:e1605. 10.1038/cddis.2014.549 25611380 PMC4669747

[B3] Chen P. Xu D. Q. Xu S. L. Xiao H. Wan S. H. Wang X. H.. (2018). Blebbistatin modulates prostatic cell growth and contrapctility through myosin II signaling. Clin. Sci. 132, 2189–2205. 10.1042/cs20180294 30279228

[B6] Coffin A. B. Rubel E. W. Raible D. W. (2013). Bax, Bcl2, and p53 differentially regulate neomycin- and gentamicin-induced hair cell death in the zebrafish lateral line. J. Assoc. Res. Otolaryngol. 14, 645–659. 10.1007/s10162-013-0404-1 23821348 PMC3767879

[B7] Durante-Mangoni E. Grammatikos A. Utili R. Falagas M. E. (2009). Do we still need the aminoglycosides? Int. J. Antimicrob. Agents 33, 201–205. 10.1016/j.ijantimicag.2008.09.001 18976888

[B9] Esterberg R. Linbo T. Pickett S. B. Wu P. Ou H. C. Rubel E. W.. (2016). Mitochondrial calcium uptake underlies ROS generation during aminoglycoside-induced hair cell death. J. Clin. Invest. 126, 3556–3566. 10.1172/jci84939 27500493 PMC5004972

[B10] Guan M. Fang Q. He Z. Li Y. Qian F. Qian X.. (2016). Inhibition of ARC decreases the survival of HEI-OC-1 cells after neomycin damage *in vitro*. Oncotarget 7, 66647–66659. 10.18632/oncotarget.11336 27556499 PMC5341827

[B11] Guo J. Chai R. Li H. Sun S. (2019). Protection of hair cells from ototoxic drug-induced hearing loss. Adv. Exp. Med. Biol. 1130, 17–36. 10.1007/978-981-13-6123-4_2 30915699

[B13] He Z. Guo L. Shu Y. Fang Q. Zhou H. Liu Y.. (2017). Autophagy protects auditory hair cells against neomycin-induced damage. Autophagy 13, 1884–1904. 10.1080/15548627.2017.1359449 28968134 PMC5788479

[B14] He Z. Sun S. Waqas M. Zhang X. Qian F. Cheng C.. (2016). Reduced TRMU expression increases the sensitivity of hair-cell-like HEI-OC-1 cells to neomycin damage *in vitro*. Sci. Rep. 6:29621. 10.1038/srep29621 27405449 PMC4942793

[B15] Hu B. H. Henderson D. Yang W. P. (2008). The impact of mitochondrial energetic dysfunction on apoptosis in outer hair cells of the cochlea following exposure to intense noise. Hear. Res. 236, 11–21. 10.1016/j.heares.2007.11.002 18082984 PMC2274916

[B16] Hu X. Weston T. A. He C. Jung R. S. Heizer P. J. Young B. D.. (2019). Release of cholesterol-rich particles from the macrophage plasma membrane during movement of filopodia and lamellipodia. Elife 8:e50231. 10.7554/eLife.50231 31486771 PMC6750930

[B17] Huang T. Cheng A. G. Stupak H. Liu W. Kim A. Staecker H.. (2000). Oxidative stress-induced apoptosis of cochlear sensory cells: otoprotective strategies. Int. J. Dev. Neurosci. 18, 259–270. 10.1016/s0736-5748(99)00094-5 10715580

[B18] Jiang M. Karasawa T. Steyger P. S. (2017). Aminoglycoside-induced cochleotoxicity: a review. Front. Cell. Neurosci. 11:308. 10.3389/fncel.2017.00308 29062271 PMC5640705

[B19] Jiang P. Ray A. Rybak L. P. Brenner M. J. (2016). Role of STAT1 and oxidative stress in gentamicin-induced hair cell death in organ of corti. Otol. Neurotol. 37, 1449–1456. 10.1097/mao.0000000000001192 27631653 PMC5125081

[B21] Kalyanaraman B. Hardy M. Podsiadly R. Cheng G. Zielonka J. (2017). Recent developments in detection of superoxide radical anion and hydrogen peroxide: opportunities, challenges, and implications in redox signaling. Arch. Biochem. Biophys. 617, 38–47. 10.1016/j.abb.2016.08.021 27590268 PMC5318280

[B22] Karasawa T. Wang Q. Fu Y. Cohen D. M. Steyger P. S. (2008). TRPV4 enhances the cellular uptake of aminoglycoside antibiotics. J. Cell Sci. 121, 2871–2879. 10.1242/jcs.023705 18682499 PMC2736053

[B23] Kovács M. Tóth J. Hetényi C. Málnási-Csizmadia A. Sellers J. R. (2004). Mechanism of blebbistatin inhibition of myosin II. J. Biol. Chem. 279, 35557–35563. 10.1074/jbc.M405319200 15205456

[B24] Lang E. Qadri S. M. Zelenak C. Gu S. Rotte A. Draeger A.. (2011). Inhibition of suicidal erythrocyte death by blebbistatin. Am. J. Physiol. Cell Physiol. 301, C490–C498. 10.1152/ajpcell.00043.2011 21593446

[B26] Li F. Fan X. Zhang Y. Zhang Y. Ma X. Kou J.. (2018). Inhibition of myosin IIA-actin interaction prevents ischemia/reperfusion induced cardiomyocytes apoptosis through modulating PINK1/Parkin pathway and mitochondrial fission. Int. J. Cardiol. 271, 211–218. 10.1016/j.ijcard.2018.04.079 30144997

[B27] Li H. Song Y. He Z. Chen X. Wu X. Li X.. (2018). Meclofenamic acid reduces reactive oxygen species accumulation and apoptosis, inhibits excessive autophagy, and protects hair cell-like HEI-OC1 cells from cisplatin-induced damage. Front. Cell. Neurosci. 12:139. 10.3389/fncel.2018.00139 29875633 PMC5974247

[B25] Li A. You D. Li W. Cui Y. He Y. Li W.. (2018). Novel compounds protect auditory hair cells against gentamycin-induced apoptosis by maintaining the expression level of H3K4me2. Drug Deliv. 25, 1033–1043. 10.1080/10717544.2018.1461277 30799660 PMC6058728

[B28] Liu L. Chen Y. Qi J. Zhang Y. He Y. Ni W.. (2016). Wnt activation protects against neomycin-induced hair cell damage in the mouse cochlea. Cell Death Dis. 7:e2136. 10.1038/cddis.2016.35 26962686 PMC4823936

[B29] Liu W. Xu X. Fan Z. Sun G. Han Y. Zhang D.. (2019). Wnt signaling activates TIGAR and protects against cisplatin-induced spiral ganglion neuron damage in the mouse cochlea. Antioxid. Redox Signal. 30, 1389–1410. 10.1089/ars.2017.7288 29587485

[B30] Liu X. Z. Yan D. (2007). Ageing and hearing loss. J. Pathol. 211, 188–197. 10.1002/path.2102 17200945

[B31] Marcotti W. van Netten S. M. Kros C. J. (2010). The aminoglycoside antibiotic dihydrostreptomycin rapidly enters mouse outer hair cells through the mechano-electrical transducer channels. J. Physiol. 567, 505–521. 10.1113/jphysiol.2005.085951 15994187 PMC1474200

[B32] Miura M. Taguchi Y. Handoh T. Hasegawa T. Takahashi Y. Morita N.. (2018). Regional increase in ROS within stretched region exacerbates arrhythmias in rat trabeculae with nonuniform contraction. Pflugers Arch. 470, 1349–1357. 10.1007/s00424-018-2152-x 29736684

[B20] Nadol J. B. Jr. (1993). Hearing loss. N. Eng. J. Med. 329, 1092–1102. 10.1056/NEJM199310073291507 8371732

[B33] Quan Y. Xia L. Shao J. Yin S. Cheng C. Y. Xia W.. (2015). Adjudin protects rodent cochlear hair cells against gentamicin ototoxicity *via* the SIRT3-ROS pathway. Sci. Rep. 5:8181. 10.1038/srep08181 25640330 PMC4313083

[B35] Samudio I. Konopleva M. Hail N. Jr. Shi Y. X. McQueen T. Hsu T.. (2005). 2-cyano-3,12-dioxooleana-1,9-dien-28-imidazolide (CDDO-Im) directly targets mitochondrial glutathione to induce apoptosis in pancreatic cancer. J. Biol. Chem. 280, 36273–36282. 10.1074/jbc.m507518200 16118208

[B36] Seidman M. D. Khan M. J. Bai U. Shirwany N. Quirk W. S. (2000). Biologic activity of mitochondrial metabolites on aging and age-related hearing loss. Am. J. Otol. 21, 161–167. 10.1016/s0196-0709(00)80003-4 10733178

[B37] Sena L. A. Chandel N. S. (2012). Physiological roles of mitochondrial reactive oxygen species. Mol. Cell 48, 158–167. 10.1016/j.molcel.2012.09.025 23102266 PMC3484374

[B38] Vicente-Manzanares M. Ma X. Adelstein R. S. Horwitz A. R. (2009). Non-muscle myosin II takes centre stage in cell adhesion and migration. Nat. Rev. Mol. Cell Biol. 10, 778–790. 10.1038/nrm2786 19851336 PMC2834236

[B39] Wang W. Y. Davidson C. D. Lin D. Baker B. M. (2019). Actomyosin contractility-dependent matrix stretch and recoil induces rapid cell migration. Nat. Commun. 10:1186. 10.1038/s41467-019-09121-0 30862791 PMC6414652

[B40] Wang X. Ling C. C. Li L. Qin Y. Qi J. Liu X.. (2016). MicroRNA-10a/10b represses a novel target gene mib1 to regulate angiogenesis. Cardiovasc. Res. 110, 140–150. 10.1093/cvr/cvw023 26825552

[B41] Wang Y. Xu Y. Liu Q. Zhang Y. Gao Z. Yin M.. (2017). Myosin IIA-related actomyosin contractility mediates oxidative stress-induced neuronal apoptosis. Front. Mol. Neurosci. 10:75. 10.3389/fnmol.2017.00075 28352215 PMC5348499

[B42] Waqas M. Sun S. Xuan C. Fang Q. Zhang X. Islam I.. (2017). Bone morphogenetic protein 4 promotes the survival and preserves the structure of flow-sorted Bhlhb5+ cochlear spiral ganglion neurons *in vitro*. Sci. Rep. 7:3506. 10.1038/s41598-017-03810-w 28615657 PMC5471210

[B43] Yoon C. Choi C. Stapleton S. Mirabella T. Howes C. Dong L.. (2019). Myosin IIA-mediated forces regulate multicellular integrity during vascular sprouting. Mol. Biol. Cell 30, 1974–1984. 10.1091/mbc.e19-02-0076 31318321 PMC6727772

[B44] Yu X. Liu W. Fan Z. Qian F. Zhang D. Han Y.. (2017). c-Myb knockdown increases the neomycin-induced damage to hair-cell-like HEI-OC1 cells *in vitro*. Sci. Rep. 7:41094. 10.1038/srep41094 28112219 PMC5253735

[B45] Zhang S. Zhang Y. Dong Y. Guo L. Zhang Z. Shao B.. (2019). Knockdown of Foxg1 in supporting cells increases the trans-differentiation of supporting cells into hair cells in the neonatal mouse cochlea. Cell. Mol. Life Sci. [Epub ahead of print]. 10.1007/s00018-019-03291-2 31485717 PMC7113235

[B46] Zhao B. Qi Z. Li Y. Wang C. Fu W. Chen Y. G. (2015). The non-mu scle-myosin-II heavy chain Myh9 mediates colitis-induced epithelium injury by restricting Lgr5+ stem cells. Nat. Commun. 6:7166. 10.1038/ncomms8166 25968904

[B47] Zimmerman E. Lahav A. (2013). Ototoxicity in preterm infants: effects of genetics, aminoglycosides, and loud environmental noise. J. Perinatol. 33, 3–8. 10.1038/jp.2012.105 22878560

