# Supplementary figures and images for: Blebbistatin Inhibits Neomycin-Induced Apoptosis in Hair Cell-Like HEI-OC-1 Cells and in Cochlear Hair Cells
Source: Front Cell Neurosci. 2020 Feb 5;13:590. doi: 10.3389/fncel.2019.00590 (PMC7025583; doi:10.3389/fncel.2019.00590)

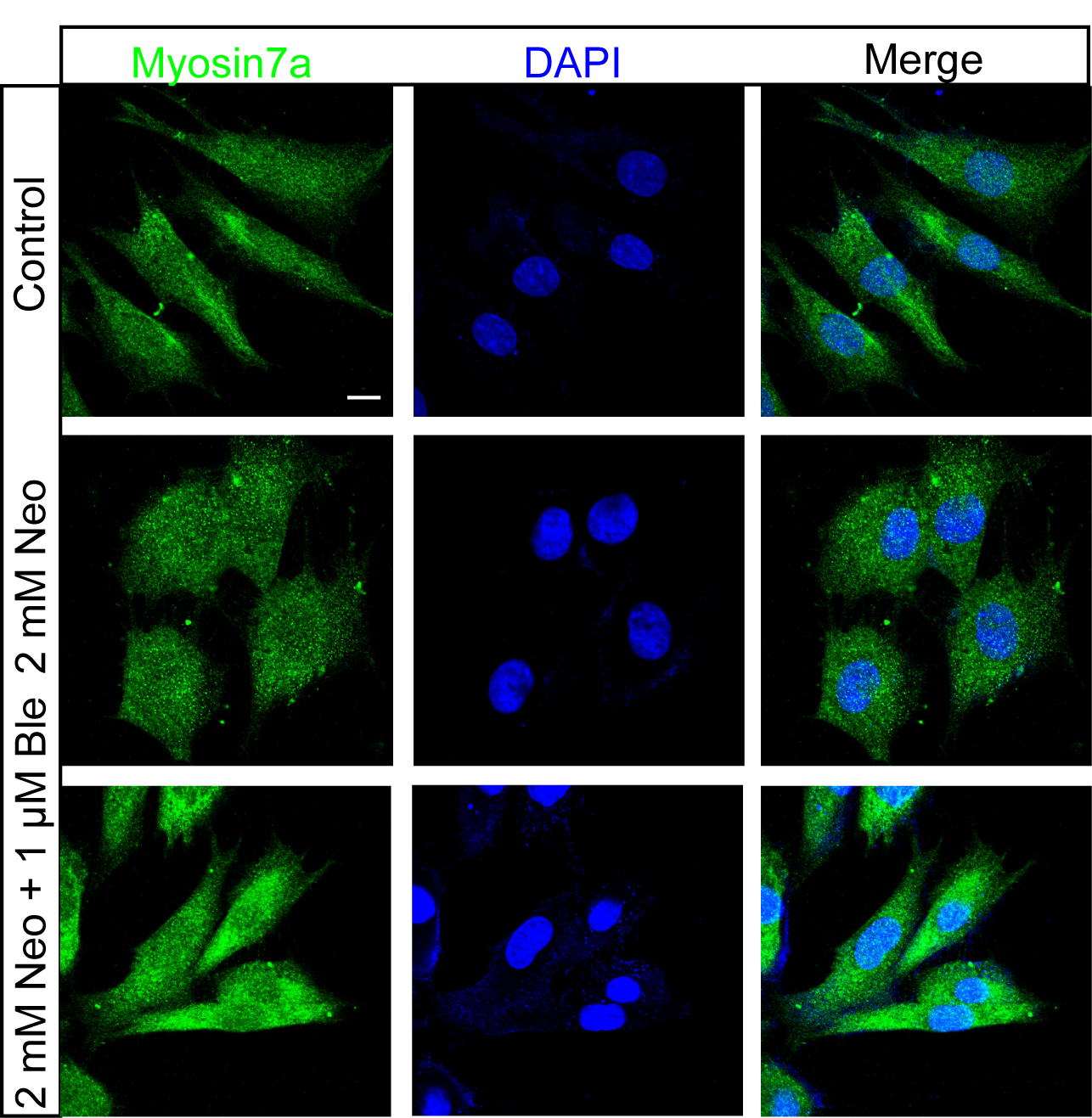

Supplement: Supplementary file 1 [file Image_1.tif]
